# Availability of living donor optimizes timing of liver transplant in high-risk waitlisted cirrhosis patients

**DOI:** 10.18632/aging.204982

**Published:** 2023-09-02

**Authors:** Fakhar Ali Qazi Arisar, Shiyi Chen, Catherine Chen, Noorulsaba Shaikh, Ravikiran Sindhuvalada Karnam, Wei Xu, Sumeet K. Asrani, Zita Galvin, Gideon Hirschfield, Keyur Patel, Cynthia Tsien, Nazia Selzner, Mark Cattral, Leslie Lilly, Mamatha Bhat

**Affiliations:** 1 Ajmera Transplant Centre, Toronto General Hospital, University Health Network, Toronto, Ontario M5G 2N2, Canada; 2 Division of Gastroenterology and Hepatology, Department of Medicine, University of Toronto, Toronto, Ontario M5G 2N2, Canada; 3 Department of Biostatistics, Princess Margaret Cancer Center, University Health Network, Toronto, Ontario M5G 2C1, Canada; 4 Division of Biostatistics, Dalla Lana School of Public Health, University of Toronto, Toronto, Ontario M5G 2C1, Canada; 5 Division of Hepatology, Department of Medicine, Baylor University Medical Center, Dallas, TX 75246, USA; 6 Toronto Centre for Liver Disease, Toronto General Hospital, University Health Network, Toronto, Ontario M5G 2C4, Canada; 7 Department of Surgery, University of Toronto, Toronto, Ontario M5G 2N2, Canada; 8 National Institute of Liver and GI Diseases, Dow University of Health Sciences, Karachi, Sindh 75330, Pakistan

**Keywords:** living donor liver transplant, frailty, old age, short-statured, MELD score, prediction model

## Abstract

Liver transplant (LT) candidates have become older and frailer, with growing Non-alcoholic steatohepatitis (NASH) and comorbid disease burden in recent years, predisposing them for poor waitlist outcomes. We aimed to evaluate the impact of access to living donor liver transplantation (LDLT) in waitlisted patients at highest risk of dropout. We reviewed all adult patients with decompensated cirrhosis listed for LT from November 2012 to December 2018. Patients with a potential living donor (pLD) available were identified. Survival analyses with Cox Proportional Hazards models and time to LT with Competing risk models were performed followed by prediction model development. Out of 860 patients who met inclusion criteria, 360 (41.8%) had a pLD identified and 496 (57.6%) underwent LT, out of which 170 (34.2%) were LDLT. The benefit of pLD was evident for all, but patients with moderate to severe frailty at listing (interaction *p* = 0.03), height <160 cm (interaction *p* = 0.03), and Model for end stage liver disease (MELD)-Na score <20 (interaction *p* < 0.0001) especially benefited. Our prediction model identified patients at highest risk of dropout while waiting for deceased donor and most benefiting of pLD (time-dependent area under the receiver operating characteristic curve 0.82). Access to LDLT in a transplant program can optimize the timing of transplant for the increasingly older, frail patient population with comorbidities who are at highest risk of dropout.

## INTRODUCTION

Mortality on the liver transplant (LT) waiting list continues to be partly driven by the disconnect between organ supply and demand. Approximately, one out of 5 patients are removed from the LT waitlist due to death or medical unsuitability [1]. Moreover, the waitlist population has changed significantly over the last decade. The mean age of newly listed patients has increased from 51.2 years to 55.7 years between 2002 and 2014 [2]. While the proportion of older (aged ≥65 years) candidates has substantially increased on waitlist from 8.9% to 20.8% over last decade [1]. Furthermore, Non-alcoholic steatohepatitis (NASH) cirrhosis has now emerged as a leading indication for liver transplantation (LT) in North America [3, 4]. NASH patients carry significant comorbid disease burden such as diabetes, hypertension, ischemic heart disease (IHD) and chronic kidney disease (CKD) which affect outcomes of cirrhosis patients [5]. They are also older and more frailer than those with other etiologies of cirrhosis, which further increases waitlist mortality and decreases the probability of transplant [2, 6–8].

The current model of organ allocation depends on assessment of medical urgency for transplant and mortality on the waiting list using MELD-Na. Due to the paucity of organ supply in relation to demand, patients must often become very sick to attract a deceased donor organ. However, given the changing dynamics of the waitlist population, the risk is that they become too sick for transplant in the process. In the setting of scarce deceased donor organs, living donor liver transplantation (LDLT) represents an important alternative. Access to LDLT shortens the median waiting time and significantly decreases waitlist morbidity and mortality for all waitlisted patients [9, 10]. However, LDLT is not widely available in North America and Europe.

We hypothesized that the evolving demographics of waitlisted patients in recent years renders them at higher risk of dropping out with prolonged waiting time, and that access to LDLT becomes especially important in this context.

## MATERIALS AND METHODS

### Study design

We retrospectively reviewed all adult patients listed for LT from November 13th, 2012, to December 31st, 2018, in the Multi-Organ Transplant Program at the University Health Network (UHN) in Toronto, Canada. Our centre performs around 200 LTs annually, of which 50–70 are LDLT. The start date was chosen, as this is when the MELD-Na system was adapted in the province of Ontario to prioritize the need of transplant while on waitlist. All patients were followed from time of listing to LT or dropout or until December 31st, 2020. In our program, NASH cirrhosis was diagnosed either based on findings of significant steatosis on pre-transplant liver biopsy or explant pathology, or presence of risk factors (diabetes, obesity, and metabolic syndrome) in the absence of significant alcohol consumption and no evidence of other etiology on serology or histopathology. Patients listed with NASH concomitant with a predominant secondary etiology of chronic liver disease were categorized under non-NASH group for purpose of analysis. Patients listed with MELD exception points for any reason, hepatoma, fulminant liver failure, combined solid organ/multi-organ transplant, or relisted for transplantation were excluded (Supplementary Figure 1).

A potential living donor (pLD) was defined as an individual who met all 3 criteria: (1) had applied with a medical history form for evaluation as a living donor, (2) was found to be appropriate for donation after the initial screening stage, and (3) had undergone imaging assessment [11, 12]. There is no difference in listing criteria for patients with or without pLD.

### Patient characteristics

Demographic and clinical characteristics at the time of listing, including age, sex, body mass index (BMI), blood group, liver disease etiology, decompensation of liver disease such as portosystemic encephalopathy, ascites, variceal bleeding, hepatorenal syndrome (HRS) and spontaneous bacterial peritonitis (SBP), comorbidities including type 2 diabetes mellitus, hypertension, IHD and CKD were documented. Functional capacity was categorized from 1 to 9 using the Clinical Frailty Scale (CFS) [13]. Patients were staged as no (CFS 1 to 3), mild (CFS 4 to 5), moderate (CFS 6) or severe frailty (CFS 7 to 9) [14]. This scoring system has been prospectively evaluated in liver disease patients [15]. All patients were followed from the time of listing to LT or dropout from the waiting list. Dropouts occurred due to death, medical unsuitability, refusal for LT, or improvement of patient to the point where transplantation was no longer required. The study was approved by the Research Ethics Board of the UHN (CAPCR ID 19-5665.0).

### Statistical methods

#### Descriptive analyses

Descriptive statistics were performed for demographic and clinical variables. Counts and proportions were calculated for categorical variables and the differences between patients with and without a potential living donor (pLD) were compared using Chi-squared test or Fisher’s exact test. Mean ± standard deviation and median (range) were calculated for continuous variables and the differences between the pLD and non-pLD group were compared using two sample *t*-tests or Wilcoxon tests, depending on the distribution of the data.

Cumulative incidence of transplant by pLD status was plotted and group differences were compared using Gray k-sample test. For the complete sample as well as a subgroup of patients who failed to receive a transplant, Kaplan-Meier plot for “time to death or delisting due to medical unsuitability was also plotted and differences between patients with and without a pLD were compared using log-rank test.

#### Competing risk and survival analyses

To examine whether patients with certain characteristics particularly benefit (with improved access to transplant) from having a pLD, nine patient characteristics of interest were identified based on clinical knowledge and previous literature identifying risk factors for waitlist mortality: age, sex, height, primary etiology, prior history of IHD, diabetes status, frailty, Na-MELD and GFR. Supplementary Table 1 shows the percentage of missing data for these variables.

For each of the 9 characteristics of interest, subgroup analyses were performed where patients were categorized into different subgroups using the cut-off associated with the characteristic’s variable. The cut-offs were selected based on sensitivity analyses. Within each subgroup, cumulative incidence of transplant was plotted comparing pLD and no pLD, while death was treated as a competing risk event. In addition, Gray’s tests were used to examine the effect of pLD on cumulative incidence of transplant. To examine whether the effects of pLD differ between different categories of the same feature, cause-specific hazard models were constructed, and variables included in the models were the characteristics variable, pLD and the characteristics × pLD interaction term. A significant interaction term (*p* < 0.05) signifies the effects of pLD differ between different categories of the characteristic’s variable, and that patients with certain characteristics particularly benefit from having a pLD and thus improved access to transplant.

Similarly, for each characteristic of interest, the consequences of not getting a transplant were examined. On non-transplanted patients, Kaplan-Meier plots on “time to death or delisting due to bad outcomes” were plotted and stratified by identified factors of interest. Cox Proportional Hazard models were built to determine the effects of these characteristics on survival among non-transplanted patients.

#### Prediction model

Multiple imputation with Markov Chain Monte Carlo method (MCMC) was used to impute missing values for frailty score, eGFR and height. Missing at random (MAR) assumption was examined, and patient characteristics did systematically differ between patients with or without frailty score, hence satisfied. The imputed dataset was then used to develop a prediction model that predicts a patient’s potential to benefit from having access to living donation. To obtain the derivation and validation sets, stratified random sampling technique was employed to split the original dataset with an 80:20 ratio. On the derivation dataset (*n* = 689), a prediction model was developed from the 9 identified features, followed by validation on the validation dataset (*n* = 171). Given the collinearity of GFR and MELD score (collineary coefficient −0.41, *p* < 0.0001), GFR was excluded from the final model. Time-dependent area under the curves (AUCs), Kaplan-Meier survival curves and cumulative incidences of transplant were plotted and compared. Calibration plots were generated for the developed model to assess prediction estimations in both the derivation dataset and validation dataset. Details of the risk score and prediction model derivation are described in the Supplementary Materials and Methods.

Statistical significance was defined as *p*-value ≤ 0.05. SAS 9.4 (SAS Institute, Cary, NC, USA) was used to perform statistical analyses.

### Data availability statement

The data that support the findings of this study are available on request from the corresponding author. The data are not publicly available due to privacy or ethical restrictions.

## RESULTS

### Patient characteristics

Out of 2191 patients listed, 860 fulfilled the inclusion criteria and were included in the final analysis (Supplementary Figure 1). The mean age of our patients was 54.6 years; 41.3% were females. 360 (41.8%) patients had a pLD identified. 63% of pLDs were approved as donors. 496 (57.6%) patients underwent LT, 170 (34.2%) were LDLT. Median time to receive a transplant was 75 (0–1725) days. Tables 1 summarize the clinical parameters and waitlist outcomes.

**Table 1:** Demographic, clinical and laboratory parameters of all patients according to pLD status.

		**Total (*n* = 860)**	**pLD**		
			**No (*n* = 500)**	**Yes (*n* = 360)**	***P* Value**
**Age at listing (years)**	Mean (SD)	54.6 (10.40)	55.1 (9.57)	53.9 (11.43)	0.10
	≥60	307 (36%)	179 (36%)	128 (36%)	0.94
**Sex**	Female	355 (41%)	178 (36%)	177 (49%)	<0.001
**Height at list (cm)**	Mean (SD)	169.6 (9.79)	170.1 (9.59)	168.8 (10.02)	0.05
	<165	251 (29%)	127 (26%)	124 (34%)	0.004
	<160	139 (16%)	70 (14%)	69 (19%)	0.04
**Weight at list (Kg)**	Mean (SD)	79.7 (19.46)	80.6 (19.25)	78.4 (19.70)	0.11
**BMI at list (Kg/m^2^)**	Mean (SD)	27.6 (5.81)	27.8 (5.76)	27.4 (5.89)	0.37
	>30	260 (30%)	152 (31%)	108 (30%)	0.86
**Primary diagnosis**	AIH	47 (6%)	24 (5%)	23 (6%)	–
	CC	34 (4%)	22 (4%)	12 (3%)	
	ALD	257 (30%)	185 (37%)	72 (20%)	
	HBV	32 (4%)	25 (5%)	7 (2%)	
	HCV	129 (15%)	86 (17%)	43 (12%)	
	NASH	176 (20%)	92 (19%)	84 (24%)	
	PBC	56 (6%)	20 (4%)	36 (10%)	
	PSC	87 (10%)	26 (5%)	61 (17%)	
	Others	42 (5%)	20 (4%)	22 (6%)	
**Comorbidities**	HTN	189 (22%)	108 (22%)	81 (22%)	0.75
	DM	219 (26%)	121 (24%)	98 (27%)	0.32
	Insulin Use	121 (14%)	65 (13%)	56 (16%)	0.29
	Dyslipidemia	108 (13%)	60 (12%)	48 (13%)	0.56
	CKD	44 (5%)	27 (5%)	17 (5%)	0.66
	IHD	53 (6%)	29 (6%)	24 (7%)	0.60
**Decompensation (at any time before end of listing)**	Encephalopathy	632 (74%)	372 (74%)	260 (72%)	0.48
	Variceal bleeding	348 (40%)	214 (43%)	134 (37%)	0.10
	Ascites	752 (87%)	443 (89%)	309 (86%)	0.23
	Paracentesis- dependent	430 (50%)	259 (52%)	171 (48%)	0.21
	SBP	178 (21%)	99 (20%)	79 (22%)	0.44
	HRS	194 (23%)	123 (25%)	071 (20%)	0.09
**Na MELD (at listing)**	Median (Range)	22.0 (6–54)	23.0 (7–54)	20.0 (6–50)	<0.001
	<20	312 (36.3%)	151 (30.2%)	161 (44.7%)	<0.001
**Na MELD (before transplant/dropout)**	Median (Range)	24.0 (6–57)	26.0 (6–50)	23.0 (6–57)	<0.001
**MDRD eGFR ml/min/1.73 m^2^**	Median (Range)	75.0 (15–120)	72.0 (15–120)	80.0 (15–120)	0.004
	<60	310 (36%)	197 (40%)	113 (31%)	0.014
**Frailty score at time of listing**	Mean (SD)	4.21 (1.45)	4.23 (1.46)	4.18 (1.43)	0.65
	Moderate to severe	145 (22%)	082 (22%)	63 (22%)	0.88
	Missing	210	136	74	
**ICU stay in last 90 days before end of listing**	*n* (%)	145 (17%)	89 (18%)	56 (16%)	0.39
**Cumulative LOS last 90 days before end of listing**	Median (Range)	1.0 (0–90)	1.0 (0.–90)	1.0 (0–90)	0.17
	Missing	95	68	27	–
**Number of hospitalizations last 90 days before end of listing**	Median (Range)	1.0 (0–16)	1.0 (0–11)	1.0 (0–16)	<0.001

Abbreviations: AIH: Autoimmune hepatitis; ALD: Alcoholic liver disease; BMI: Body mass index; CC: Cryptogenic cirrhosis; CKD: Chronic kidney disease; DM: Diabetes mellitus; eGFR: Estimated glomerular filtration rate; HBV: Hepatitis B virus; HCV: Hepatitis C virus; HRS: Hepatorenal syndrome; HTN: Hypertension; ICU: Intensive care unit; IHD: Ischemic heart disease; LOS: Length of stay; NASH: Non-alcoholic steatohepatitis; PBC: Primary biliary cholangitis; pLD: Potential living donor; PSC: Primary sclerosing cholangitis; SBP: Spontaneous bacterial peritonitis; SD: Standard deviation.

### Clinical characteristics of patients listed with pLD

Patients listed with a pLD had lower Na-MELD scores (20 (6–50) vs. 23 (7–54); *p* < 0.001), higher rate of transplant (74.4% vs. 45.6%; *p* < 0.001), more female (49.2% vs. 35.6%; *p* < 0.001) and had height <160 cm (19.2% vs. 14.1%; *p* = 0.04). Tables 1, 2 and Supplementary Table 2 describes the basic demographics. Having a pLD was protective against death or dropout due to medical unsuitability (52% vs. 24%; *p* < 0.001) (Table 3).

**Table 2:** Demographic, clinical and laboratory parameters of all patients according to transplant status.

		**Total (*n* = 860)**	**Transplanted**		
			**No (*n* = 364)**	**Yes (*n* = 496)**	***P* value**
**Age at listing (years)**	Mean (SD)	54.6 (10.40)	56.7 (8.98)	53.1 (11.09)	<0.001
	≥60	307 (36%)	154 (42%)	153 (31%)	<0.001
**Sex**	Female	355 (41%)	147 (40%)	208 (42%)	0.65
**Height at list (cm)**	Mean (SD)	169.6 (9.79)	169.1 (10.20)	169.9 (9.48)	0.24
	<165	251 (29%)	112 (31%)	139 (28%)	0.35
	<160	139 (16%)	67 (19%)	72 (15%)	0.12
**Weight at list (Kg)**	Mean (SD)	79.7 (19.46)	79.7 (20.70)	79.6 (18.52)	0.94
**BMI at list (Kg/m^2^)**	Mean (SD)	27.6 (5.81)	27.8 (6.04)	27.5 (5.65)	0.50
	>30	260 (30%)	114 (32%)	146 (30%)	0.50
**Primary diagnosis**	AIH	47 (6%)	19 (5%)	28 (6%)	–
	CC	34 (4%)	21 (6%)	13 (3%)	
	ALD	257 (30%)	130 (37%)	127 (26%)	
	HBV	32 (4%)	12 (3%)	20 (4%)	
	HCV	129 (15%)	67 (18%)	62 (12%)	
	NASH	176 (20%)	65 (18%)	111 (22%)	
	PBC	56 (6%)	19 (5%)	37 (7%)	
	PSC	87 (10%)	19 (5%)	68 (14%)	
	Others	42 (5%)	12 (3%)	30 (6%)	
**Comorbidities**	HTN	189 (22%)	79 (22%)	110 (22%)	0.87
	DM	219 (26%)	94 (26%)	125 (25%)	0.84
	Insulin Use	121 (14%)	50 (14%)	71 (14%)	0.81
	Dyslipidemia	108 (13%)	50 (14%)	58 (12%)	0.37
	CKD	44 (5%)	21 (6%)	23 (5%)	0.46
	IHD	53 (6%)	26 (7%)	27 (5%)	0.31
**Decompensation (at any time before end of listing)**	Encephalopathy	632 (74%)	262 (72%)	370 (75%)	0.39
	Variceal bleeding	348 (40%)	150 (41%)	198 (40%)	0.70
	Ascites	752 (87%)	323 (89%)	429 (86%)	0.33
	Paracentesis- dependent	430 (50%)	198 (54%)	232 (47%)	0.027
	SBP	178 (21%)	84 (23%)	94 (19%)	0.14
	HRS	194 (23%)	075 (21%)	119 (24%)	0.24
**Na MELD (at listing)**	Median (Range)	22.0 (6–54)	20.5 (7 – 54)	23.0 (6–50)	<0.001
	<20	312 (36.3%)	161 (44.2%)	151 (30.4%)	<0.001
**Na MELD (before transplant/dropout)**	Median (Range)	24.0 (6–57)	22.0 (7–57)	26.0 (6–51)	<0.001
**MDRD eGFR ml/min/1.73 m^2^**	Median (Range)	75.0 (15–120)	74.0 (15–120)	77.5 (15–120)	0.09
	<60	310 (36%)	137 (38%)	173 (35%)	0.37
**Frailty score at time of listing**	Mean (SD)	4.21 (1.45)	4.41 (1.54)	4.06 (1.35)	0.002
	Moderate to severe	145 (22%)	80 (28%)	65 (18%)	0.001
	Missing	210	81	129	
**ICU stay in last 90 days before end of listing**	*n* (%)	145 (17%)	82 (22%)	63 (13%)	<0.001
**Cumulative LOS last 90 days before end of listing**	Median (Range)	1.0 (0–90)	0.0 (0–90)	2.0 (0–90)	<0.001
	Missing	95	54	41	–
**Number of hospitalizations last 90 days before end of listing**	Median (Range)	1.0 (0–16)	0.0 (0–10)	1.0 (0–16)	<0.001

Abbreviations: AIH: Autoimmune hepatitis; ALD: Alcoholic liver disease; BMI: Body mass index; CC: Cryptogenic cirrhosis; CKD: Chronic kidney disease; DM: Diabetes mellitus; eGFR: Estimated glomerular filtration rate; HBV: Hepatitis B virus; HCV: Hepatitis C virus; HRS: Hepatorenal syndrome; HTN: Hypertension; ICU: Intensive care unit; IHD: Ischemic heart disease; LOS: Length of stay; NASH: Non-alcoholic steatohepatitis; PBC: Primary biliary cholangitis; pLD: Potential living donor; PSC: Primary sclerosing cholangitis; SBP: Spontaneous bacterial peritonitis; SD: Standard deviation.

**Table 3:** Waitlist outcomes of all patients.

		**Total (*N* = 860)**	**pLD**			**Transplanted**		
			**No (*N* = 500)**	**Yes (*N* = 360)**	***P* Value**	**No (*N* = 364)**	**Yes (*N* = 496)**	***P* value**
**Time on waitlist (Days)**	Median (Range)	103 (0–1903)	97 (0–1903)	106.5 (3–1819)	0.14	215 (3–1903)	75 (0–1725)	<0.001
**Time to receive transplant (Days)**	Median (Range)	75 (0–1725)	28 (0–1511)	94 (4–1725)	<0.001	–	74.5 (0–1725)	–
**pLD**	Yes	360 (42%)	–	–	–	92 (25%)	268 (54%)	<0.001
**Outcome**	Active Listing	20 (2%)	14 (3%)	6 (2%)	<0.001	20 (6%)	0	<0.001
	De-listed	147 (17%)	120 (24%)	27 (8%)		147 (40%)	0	
	Died	197 (23%)	138 (28%)	59 (16%)		197 (54%)	0	
	Transplant	496 (58%)	228 (45%)	268 (74%)		0	496 (100%)	
**Type of Liver Transplant**	DDLT	326 (66%)	226 (99%)	100 (37%)	<0.001	0	326 (66%)	–
	LDLT	170 (34%)	2 (1%)	168 (63%)		0	170 (34%)	

Abbreviations: DDLT: Deceased donor liver transplant; LDLT: Living donor liver transplant; NASH: Non-alcoholic steatohepatitis; pLD: Potential living donor; SD: Standard deviation.

### Competing risk analysis of access to liver transplant

**Figure 1:**
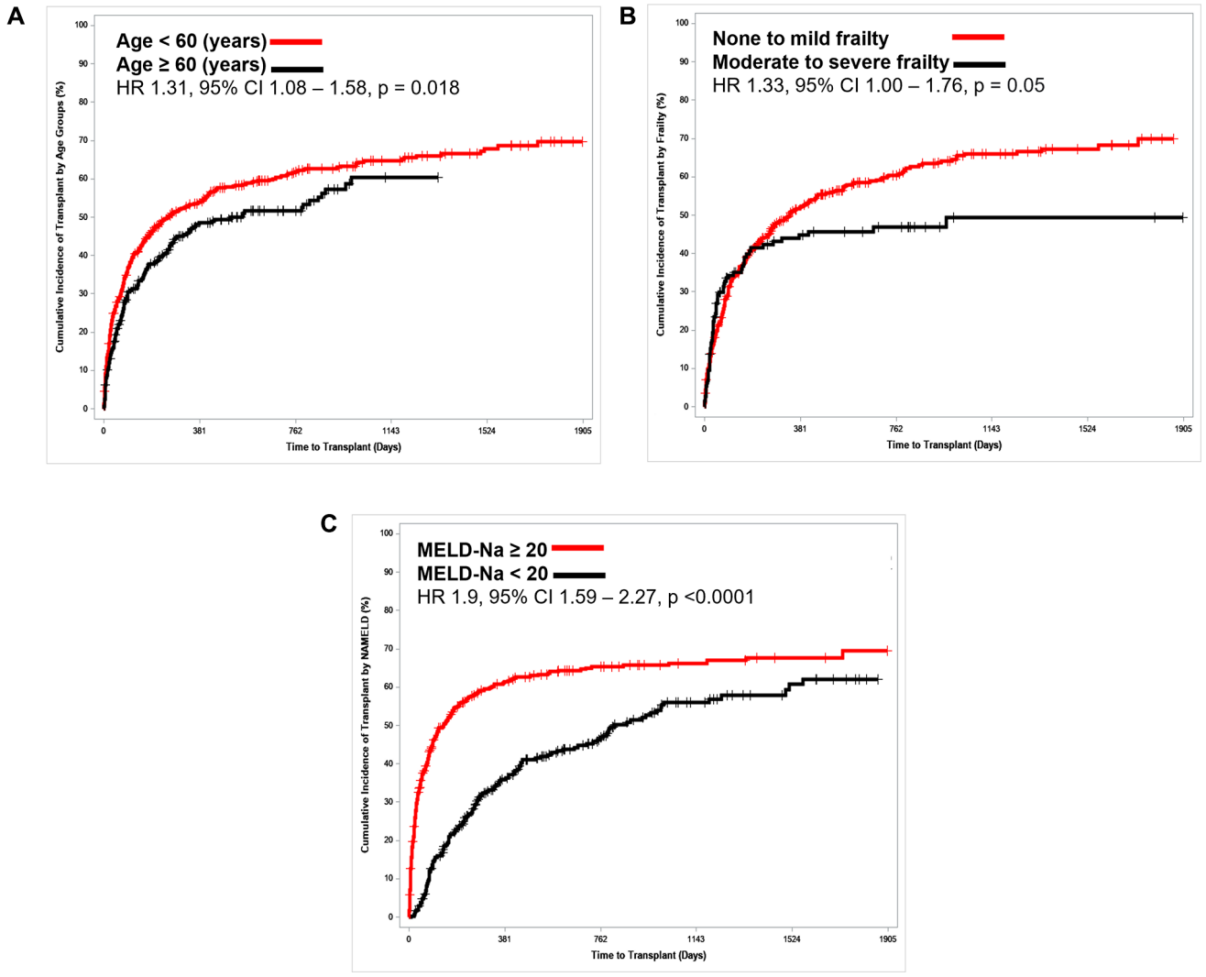
**Competing risk analysis for time to transplant stratified.** (**A**) Age, (**B**) Frailty and (**C**) MELD-Na.

Cumulative incidence of transplant in NASH and non-NASH patients was similar (HR = 1.10 (95% CI = 0.90–1.34), *p* = 0.39). Higher instantaneous rate of transplant (higher probability of having a transplant at any given time point) was observed in patients with age <60 (HR: 1.31 (95% CI: 1.08–1.58), *p* = 0.019), MELD-Na >20 (HR: 1.9 (95% CI: 1.59–2.27), *p* < 0.0001), and no/mild frailty (HR: 1.33 (95% CI: 1.00–1.76), *p* = 0.05) (Figure 1). A trend was seen for height >160 cm (HR: 1.25 (95% CI: 0.99–1.59), *p* = 0.08). However, no impact of eGFR, sex, obesity, presence of DM or history of IHD was seen on rate of transplant.

### Waitlist outcomes

Patients who either died or were delisted, were older (56.7 ± 8.98 vs. 53.1 ± 11.09 years; *p* < 0.001), moderate to severely frail (28.3% vs. 17.7%; *p* = 0.001), and had longer waitlist time (215 vs. 75 days, *p* < 0.001) (Tables 2, 3).

**Figure 2:**
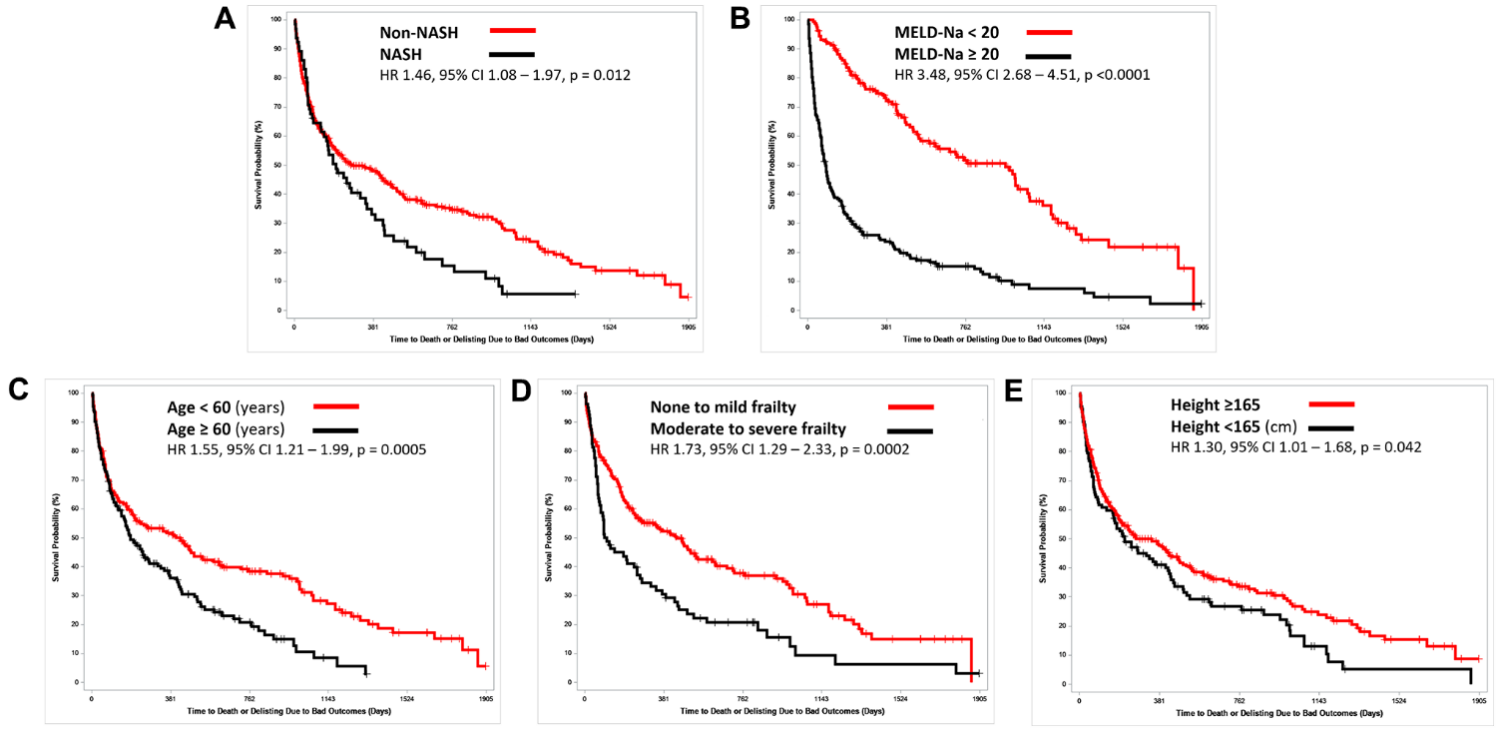
**Kaplan-Meier – Overall Survival: time to death or delisting of non-transplanted patients stratified.** (**A**) Etiology of liver disease, (**B**) MELD-Na, (**C**) Age, (**D**) Frailty, (**E**) Height.

High waitlist mortality/dropout was seen in patients with NASH etiology (HR: 1.46 (95% CI: 1.08–1.97), *p* = 0.01), age >60 (HR: 1.55 (95% CI: 1.21–1.99), *p* = 0.0005), MELD-Na >20 (HR: 3.48 (95% CI: 2.68–4.51), *p* < 0.0001), eGFR <60 (HR: 2.15 (95% CI: 1.68–2.75)), *p* < 0.0001), height <165 (HR: 1.30 (95% CI: 1.01–1.68), *p* = 0.04), and moderate to severe frailty (HR: 1.73 (95% CI: 1.29–2.33), *p* = 0.0002) (Figure 2). No difference was observed in sex, height <160 cm, obesity, presence of DM or IHD.

### Interaction of pLD with risk factors

**Figure 3:**
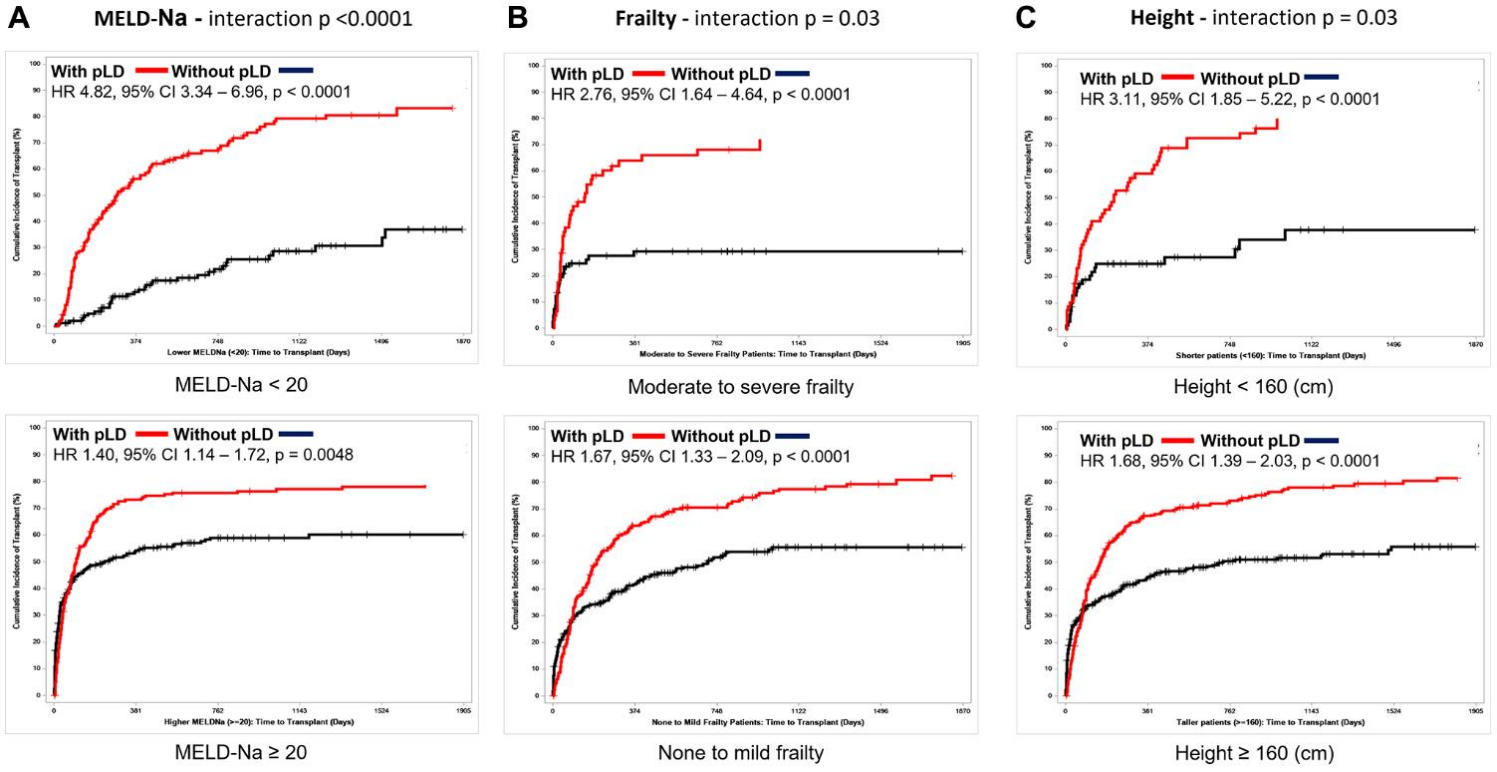
**Competing risk analysis for time to transplant stratified by availability of potential living donor for patients.** (**A**) MELD-Na <20 vs. MELD-Na ≥20; interaction *p* < 0.0001, (**B**) Moderate to severe frailty vs. None to mild frailty; interaction *p* = 0.03, and (**C**) Height <160 cm vs. ≥160 cm; interaction *p* = 0.03.

Having a pLD was associated with a higher instantaneous rate of receiving a transplant for both NASH (HR = 1.59 (95% CI = 1.09–2.31), *p* = 0.026) and non-NASH (HR = 1.84 (95% CI = 1.51–2.25), *p* < 0.0001) waitlisted cirrhosis patients. Although the magnitude of effect of pLD seems larger in non-NASH patients, there was no statistically significant difference in the effect of pLD in the two groups (interaction *p* = 0.35). Similarly, the benefit of pLD was evident regardless of age, sex, obesity, and presence of DM or IHD, but patients with MELD-Na <20 (interaction *p* < 0.0001), moderate to severe frailty (interaction *p* = 0.03), and height <160 cm (interaction *p* = 0.03) especially benefited (Figure 3 and Supplementary Table 3).

### Prediction model

We derived a prediction model using cause-specific hazard modelling as described in supplementary methods to identify patients specifically benefitting from pLD. Below is the formula derived to calculate the prediction model:

Prediction model = (−0.17452) × NASH + (−0.00776) × Age + 0.41977 × sex + 0.08806 × DM + 0.35825 × IHD + (−0.07902) × MELD-NA + (0.11491) × Frailty + (−0.03905) × Height.

**Figure 4:**
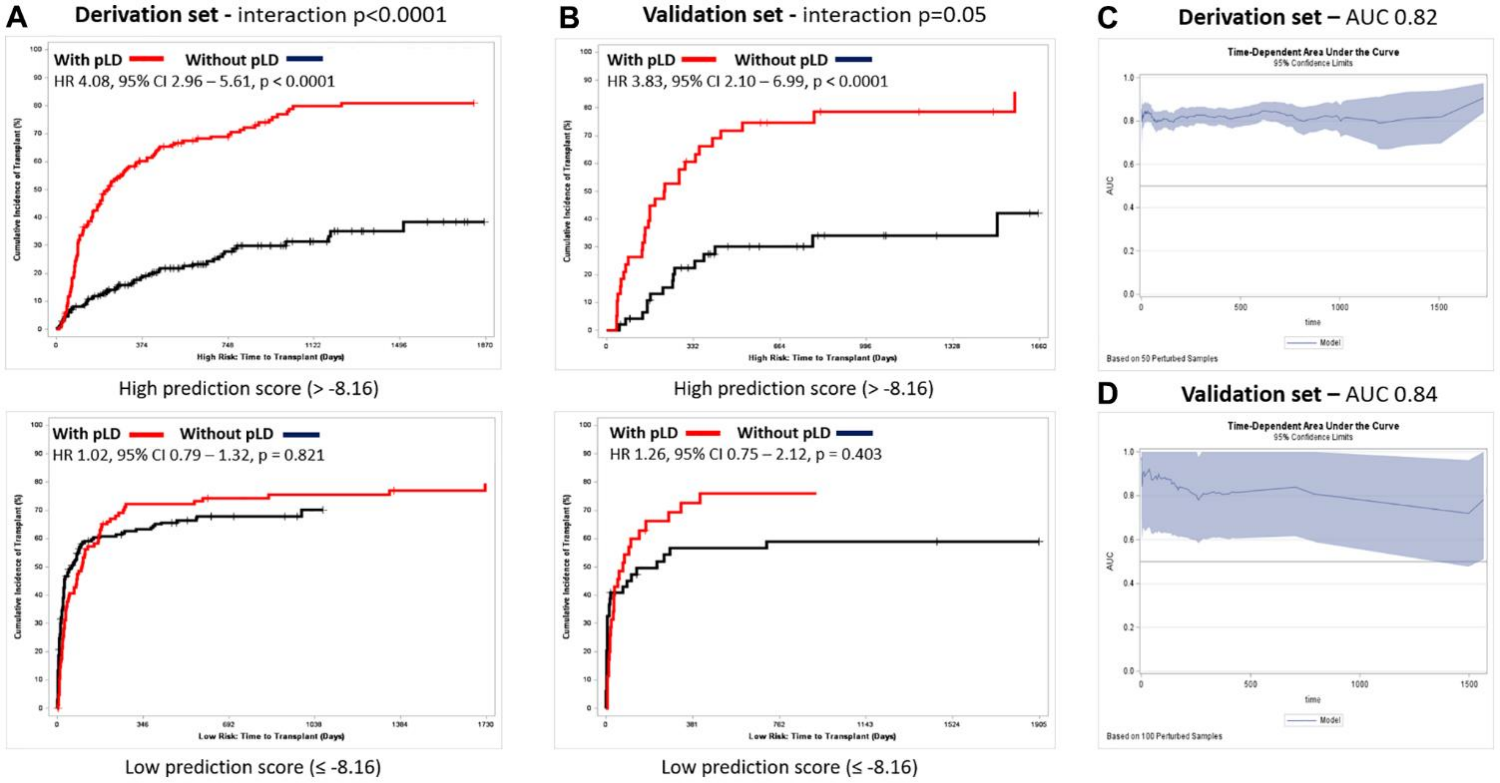
**Prediction model performance.** (**A**) Competing risk analysis for time to transplant stratified by availability of potential living donor for patients with Prediction score low vs. high on derivation set; interaction *p* < 0.0001. (**B**) Competing risk analysis for time to transplant stratified by availability of potential living donor for patients with Prediction score low vs. high on validation set; interaction *p* = 0.05. (**C**) Prediction Model area under curve on derivation set (0.82). (**D**) Prediction Model area under curve on validation set (0.84).

The cut-off score was −8.16. On the derivation set using the cause-specific hazard model, pLD was significantly associated with increased cumulative incidence of transplant in patients with high prediction score (>> −8.16) (HR 4.08 (2.96–5.62), *p* < 0.0001) (Supplementary Table 3). In patients with low prediction score (≤ −8.16), having pLD was not associated with any difference in rate/time to transplant (*p* = 0.89) (Supplementary Table 3). The interaction was significant, indicating the effect of having a pLD differed significantly by prediction score level (group *p* < 0.0001) (Supplementary Table 3 and Figure 4). The AUCs of the prediction model were 0.82 and 0.84 in the derivation and validation datasets respectively (Figure 4). The model’s calibration is provided in Supplementary Figure 2.

## DISCUSSION

Our study identifies that certain patient subgroups (short stature, MELD <20, and moderate to severe frailty) are at the highest risk for waitlist mortality with prolonged waiting time for a deceased donor organ offer. These patient subgroups, which represent a growing share of the waitlist population in recent years, would be especially protected against death or delisting if they had access to living donation at the time of listing. Certainly, LDLT is beneficial to all, with improved waitlist mortality and post-transplant outcomes.

The recent years have seen a significant increase in NASH as indication for transplant, and increasing age, frailty, and metabolic comorbidities among candidates. Moreover, NASH patients tend to have lower MELD-Na scores, slower progression of disease, [16, 17] and carry higher risk of waitlist mortality [18]. The 1-year survival on the waitlist for NASH-related cirrhosis patients have dropped from 42.8% to 25.6% over the last decade, and they are less likely to attract a deceased donor organ within the initial 90 days of listing [3]. In a recent large study based on SRTR data, NASH etiology was significantly associated with waitlist mortality [19]. In our study, the cumulative incidence of transplant was similar in NASH and non-NASH patients, though we confirmed the earlier findings of higher waitlist mortality for NASH patients as compared to non-NASH.

Frailty is associated with high waitlist mortality, [20, 21] especially in patients older than 65 years of age, [22] and independent of encephalopathy or ascites [23]. In a retrospective analysis, a higher frailty score was associated with an increased risk of delisting in NASH patients (HR1.46 (CI 1.06–2.03), *p* = 0.02) [7]. A recent multicenter study showed association of frailty with higher risk of waitlist mortality independent of age [24]. In our study, patients with none to mild frailty tended to have higher instantaneous rate of transplant while moderate to severely frail patients suffered significantly higher waitlist mortality and benefited from access to living donation.

Females are disadvantaged by the MELD scoring system for various reasons, including but not limited to low muscle mass and serum creatinine. Height also contributes to this sex disparity [25, 26]. One possible reasoning behind this occurrence is that people who are of short stature require smaller organs, which are mostly allocated to children. As a result, shorter individuals at the top of the waiting list for liver transplantation may have to wait longer to receive a liver that is a suitable size and fit for their body. Given the fact that women are shorter as compare to men, this increased mortality in shorter patients is the main driver of gender disparity in waitlist mortality [25]. We have previously shown that females can overcome this allocation inequity with access to living donation [27]. In current study we used a subset of same data by excluding all HCC patients. Although we did not find any direct impact of sex on rate of transplant, short-statured patients had a trend towards inferior transplant rate and significantly higher waitlist mortality (for height <165 cm) and significant benefit from pLD (for height <160 cm). This again supports the previous findings of high mortality/delisting (28% vs. 24%, *p* < 0.01), low transplant rates (38% vs. 44% *p* < 0.01), and 8% increased risk of waitlist mortality after adjustment for clinical and demographic characteristics (*P* < .01) in short-statured patients [28]. Furthermore, granting an extra 1, 2 MELD points to the shortest 8% of liver transplant (LT) candidates could potentially improve waitlist outcomes for female candidates [29].

High Na-MELD score is associated with increased risk of waitlist removal due to mortality or deterioration in medical condition [2]. The discrepancy between the supply and demand of deceased donor organs has resulted in longer waiting times and high waitlist mortality. To attract an organ, patients need to have high MELD score, but are at risk of becoming too sick/frail for transplant. Access to LDLT not only shortens the median waiting time and thereby significantly decreases waitlist morbidity and mortality, [9, 10] but also provides the opportunity to transplant patients earlier in their disease course while they are still fit enough to undergo transplant.

Our study clearly showed that all patients benefit from access to living donation, but pLD specifically increases the chances of getting a liver transplant and at a faster rate for the vulnerable groups i.e., frail, short stature, and low Na-MELD score. We also created a prediction model to highlight the benefit of pLD for these specific subgroups with good AUCs of ≥0.8 in both testing and validation sets. For patients with score less than −8.16, whether they get a pLD or not does not affect their potential to access transplant. For patients with score higher than −8.16, having a pLD significantly increases access to LT compared to if they do not have a pLD. These individuals might otherwise have a prolonged wait for a deceased donor offer and either die or drop off the waitlist. Table 4 describes examples of four patients having high prediction score with and without pLD, where our prediction model accurately predicted their outcome. This unique prediction model can help clinicians to identify these high-risk patients and refer them for living donation on a priority basis to a centre that performs LDLT.

**Table 4:** Patient examples.

	**Patient 1**	**Patient 2**	**Patient 3**	**Patient 4**
Age at listing (years)	64	66	66	64
Sex	Male	Male	Female	Female
Blood group	A	A	O	B
Etiology	Alcohol	NASH	PBC	Cryptogenic cirrhosis
Height (cm)	157	168	157	157
Weight (Kg)	72.5	101.1	84	60
BMI (kg/m^2^)	29.4	35.8	34	24
Hypertension	Yes	No	No	No
Diabetes	Yes	Yes	No	No
CKD	No	Yes	No	No
IHD	No	No	No	No
Encephalopathy	Yes	Yes	Yes	No
Variceal bleed	No	No	Yes	No
Ascites	Yes	Yes	Yes	No
SBP	Yes	No	No	No
HRS	No	No	No	No
Na-MELD at listing	18	11	33	33
eGFR at listing (ml/min/1.73 m^2^)	79	57	89	72
Clinical Frailty Score at listing	4	7	6	2
Frailty group at listing	None to mild	Moderate to severe	Moderate to severe	None to mild
Prediction score	−8.12	−7.71	−9.85	−10.11
Prediction score group	High	High	Low	Low
pLD	No	Yes	No	No
Time on waiting list (days)	1184	159	25	14
Outcome	Death	LDLT	Death	Death

Abbreviations: NASH: Nonalcoholic steatohepatitis; CKD: Chronic kidney disease; IHD: Ischemic heart disease; SBP: Spontaneous bacterial peritonitis; HRS: Hepatorenal syndrome; eGFR: Estimated glomerular filtration rate; pLD: Potential living donor; LDLT: Living donor liver transplant.

### Study limitations

The principal limitations of our study were that it was single center. However, being one of the largest transplant centers in North America, despite using extensive exclusion criteria, we ended up having enough patients to create and validate a prediction model. Moreover, such a study is not possible to perform with the SRTR or other such large transplant databases, as information regarding the availability of a pLD for a specific patient is not available. These registries only contain the information whether a patient ultimately received a living donor versus deceased donor transplant. It should also be noted that our study findings pertain only to those decompensated cirrhosis patients who were deemed to be suitable candidates for transplant. Certainly, many patients may not be listed for transplant due to the presence of significant comorbidities that represent a contraindication. We also acknowledge that the clinical frailty score may not be optimal as an assessment of frailty, however the retrospective nature of our study prevented the use of more robust tools such as the Liver Frailty Index. While no difference in rate of transplant was observed for low-risk patients, we do not advocate against the use of living donation in this group. One should also keep in mind the other LDLT specific issues such as size matching and patient sickness level, where LDLT may not be a feasible option. Moreover, further studies would be required to validate our prediction model externally in a more heterogenous group of patients.

## CONCLUSION

Both NASH and non-NASH cirrhosis patients on the waitlist benefit from access to a living donor, by optimizing the timing of transplant for the subgroups identified (moderate to severe frailty, short stature, and MELD-Na <20). Our model could be used to guide referral of such high-risk subgroup patients to LDLT centres earlier in their course and save more lives.

## Supplementary Materials

Supplementary Materials and Methods

Supplementary Figures

Supplementary Tables
