# Supplementary material for: Availability of living donor optimizes timing of liver transplant in high-risk waitlisted cirrhosis patients
Source: Aging (Albany NY). 2023 Sep 2;15(17):8594–612. doi: 10.18632/aging.204982 (PMC10522397; doi:10.18632/aging.204982)
Supplement: Supplementary Materials and Methods [file aging-15-204982-s001.pdf]

SUPPLEMENTARY MATERIALS AND METHODS

Prediction model

The coefficient estimations of the interaction terms ($\beta_{pLD \times feature}$) were obtained from the multivariable Cause-specific hazard model:

$F(t|x) = \beta_{pLD} \times pLD + (\beta_{age} \times age + \beta_{gender} \times gender + \beta_{height} \times height + \beta_{NASH} \times NASH + \beta_{MELD\text{-}Na} \times MELD\text{-}Na + \beta_{eGFR} \times eGFR + \beta_{DM} \times DM + \beta_{IDH} \times IHD + \beta_{frailty} \times Frailty) + (\beta_{pLD \times age}\ pLD \times Age + \beta_{pLD \times gender}\ pLD \times Gender + + \beta_{pLD \times height}\ pLD \times Height + \beta_{pLD \times NASH}\ pLD \times NASH + \beta_{pLD \times MELD\text{-}NA}\ pLD \times MELD\text{-}Na + \beta_{pLD \times eGFR}\ pLD \times eGFR + \beta_{pLD \times DM}\ pLD \times DM + \beta_{pLD \times IHD}\ pLD \times IHD + \beta_{pLD \times frailty} pLD \times Frailty)$ [1].

Next, a score was calculated for each patient by multiplying the coefficient estimations with patient's unique variable values, and taking the sum of these items:

$\text{Prediction score}_j = \beta_{pLD \times age} \times age_j + \beta_{pLD \times gender} \times gender_j + \beta_{pLD \times height} \times height_j + \beta_{pLD \times NASH} \times NASH + \beta_{pLD \times MELD\text{-}Na} \times MELD\text{-}Na_j + \beta_{pLD \times eGFR} \times eGFR_j + \beta_{pLD \times DM} \times DM_j + \beta_{pLD \times IHD} \times IDH_j + \beta_{pLD \times NAMELD} \times NAMELD_j + \beta_{pLD \times frailty} \times Frailty$. (j = patient 1 – 860) [2].

After that, a new Cause-specific hazard model (prediction model) was constructed including the prediction score, pLD status, prediction score and pLD interaction term: $F(t|x)$ = Prediction score + pLD + prediction score × pLD [3].

The time-dependent AUC of this model was calculated and plotted. Moreover, the maximally selected rank statistic method was used to determine an optimal cut-point for the prediction score. (1) Cumulative incidences of transplant were plotted and stratified by pLD, in low and high prediction score groups respectively. On non-transplanted patients, Kaplan-Meier plots on "time to death or delisting due to bad outcomes" was plotted and stratified by the binary prediction score. Cox Proportional Hazard models were built to determine the effects of prediction score on survival among non-transplanted patients. Transparent reporting of a multivariable prediction model for individual prognosis or diagnosis (TRIPOD) statement and steps outlined in the development and reporting consensus guidance were followed during our model development [2].

Validation

Internal validation of the accuracy of the prediction model was carried out on the derivation dataset. Leave-one-out bootstrap method was used to assess the internal validity of the prediction score, with 100 bootstrap samples. (3) Time-dependent AUC was generated and plotted.

External validation of the model was performed on the validation dataset ($n = 171$). For each patient, a prediction score was calculated by multiplying patient's unique feature values with corresponding coefficient estimations obtained from the derivation dataset (taken from model [2]). Similarly, a cause-specific hazard model was constructed: $F(t|x)$ = Prediction score + pLD + prediction score × pLD. Time-dependent AUC of this model was calculated and plotted. Taking the same optimal cut off point obtained in the derivation dataset and applied it to the validation set, cumulative incidences of transplant were again plotted and compared. Calibration plots were generated for the developed model to assess prediction estimations in both the derivation dataset and validation dataset. In addition, calibration plots for the model without interaction term were also plotted and compared. The calibration plot of our model also performs better than the model without the interaction term, suggesting the interaction term not only provides meaningful indication of pLD benefit, but also improves model prediction estimations.

SUPPLEMENTARY REFERENCES

1. Manchon P, Bachelet D, Francoz C, Durand F, Laouénan C. Determine an optimal cut-point in presence of competing risks to categorize a continuous distribution: An application in studying outcomes of patients with cirrhosis listed for liver transplantation. Revue d'Épidémiologie et de Santé Publique. 2020; 68:S127–8. https://doi.org/10.1016/j.respe.2020.03.056

2. Leisman DE, Harhay MO, Lederer DJ, Abramson M, Adjei AA, Bakker J, Ballas ZK, Barreiro E, Bell SC, Bellomo R, Bernstein JA, Branson RD, Brusasco V, et al. Development and Reporting of Prediction Models: Guidance for Authors From Editors of Respiratory, Sleep, and Critical Care Journals. Crit Care Med. 2020; 48:623–33. https://doi.org/10.1097/CCM.0000000000004246 PMID:32141923

3. Efron B, Tibshirani RJ. An introduction to the bootstrap: CRC press. 1994. https://doi.org/10.1201/9780429246593
