# Supplementary material for: Availability of living donor optimizes timing of liver transplant in high-risk waitlisted cirrhosis patients
Source: Aging (Albany NY). 2023 Sep 2;15(17):8594–612. doi: 10.18632/aging.204982 (PMC10522397; doi:10.18632/aging.204982)
Supplement: Supplementary Figures [file aging-15-204982-s002.pdf]

SUPPLEMENTARY FIGURES

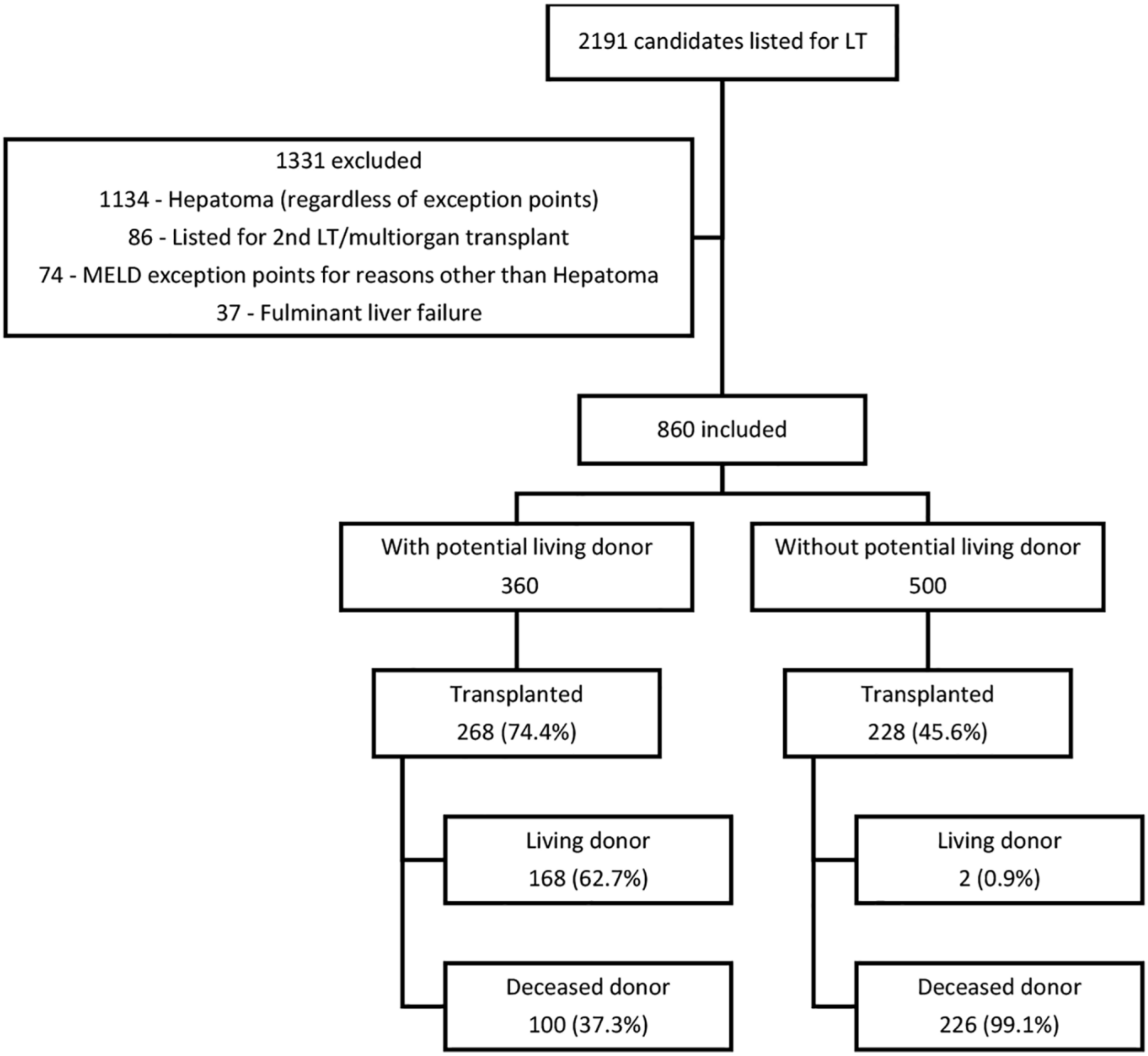

Supplementary Figure 1. Consort diagram showing study criteria and patient population.

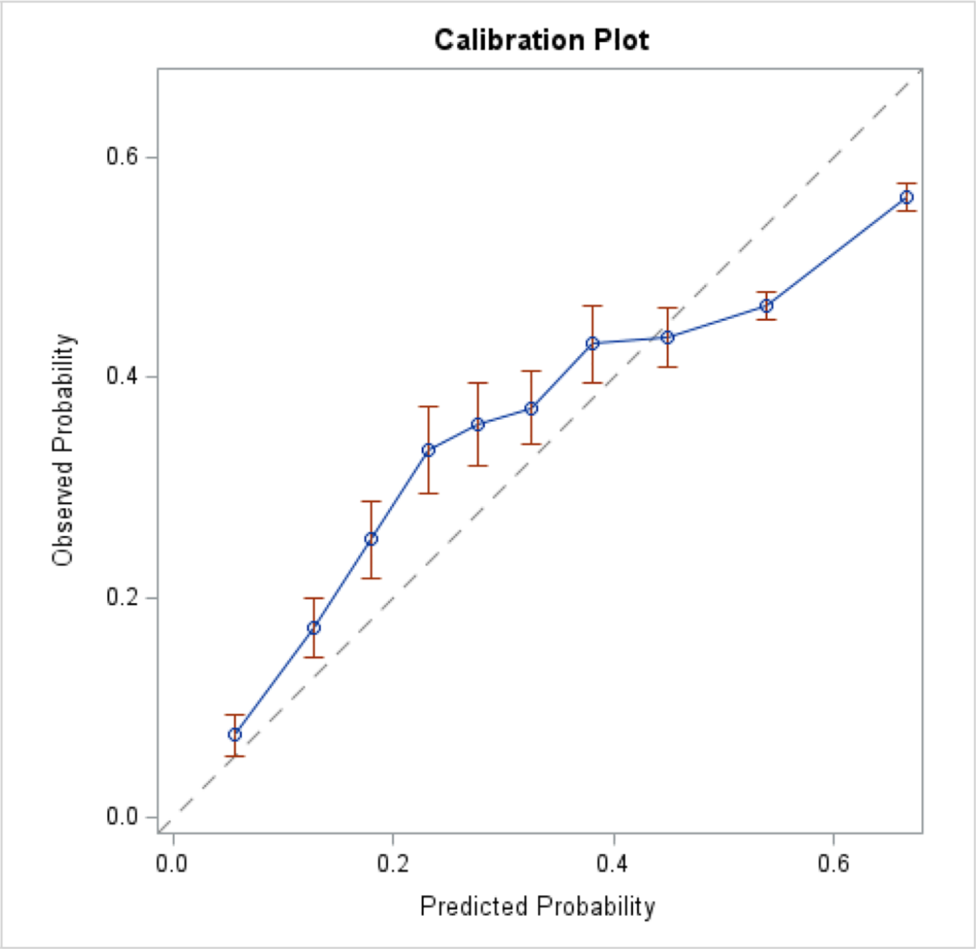

Supplementary Figure 2. Calibration plot (X-axis: Predicted probability of transplant based on the model. Y-axis: Observed probability of transplant).
