# Supplementary material for: Availability of living donor optimizes timing of liver transplant in high-risk waitlisted cirrhosis patients
Source: Aging (Albany NY). 2023 Sep 2;15(17):8594–612. doi: 10.18632/aging.204982 (PMC10522397; doi:10.18632/aging.204982)
Supplement: Supplementary Tables [file aging-15-204982-s003.pdf]

SUPPLEMENTARY TABLES

Supplementary Table 1. Percentage of missing data for the variables in the model.

Variable	*n* (%)
**Age at list**	0 (0%)
**Na MELD (at listing)**	0 (0%)
**MDRD eGFR**	2 (0.23%)
**Height at list**	4 (0.47%)
**Weight at list**	2 (0.23%)
**BMI**	5 (0.58%)
**Frailty score**	210 (24.42%)
**Sex**	0 (0%)
**DM**	0 (0%)
**IHD**	0 (0%)

Supplementary Table 2. Demographic, clinical and laboratory parameters according to cohorts used for prediction score derivation.

		Prediction score (Derivation set)				Prediction score (Validation set)			
		Total (*N* = 689)	≤ −8.16 (*N* = 307)	> −8.16 (*N* = 382)	*P* value	Total (*N* = 171)	≤ −8.16 (*N* = 84)	> −8.16 (*N* = 87)	*P* value
Age at listing (years)	Mean (SD)	54.59 (10.47)	54.33 (10.20)	54.80 (10.69)	0.56	54.76 (10.17)	54.47 (10.22)	55.04 (10.17)	0.72
	≥60	247 (36%)	101 (32%)	146 (38%)	0.15	60 (35%)	29 (35%)	31 (36%)	0.88
Gender	Female	285 (41%)	116 (38%)	169 (44%)	0.09	70 (41%)	33 (39 %)	37 (43%)	0.67
Height at list (cm)	Mean (SD)	169.43 (9.89)	172.61 (9.45)	166.89 (9.49)	<0.001	170.02 (9.41)	172.97 (9.31)	167.18 (8.63)	<0.001
Weight at list (Kg)	Mean (SD)	79.42 (19.67)	82.77 (20.31)	76.76 (18.76)	<0.001	80.72 (18.59)	84.67 (19.79)	76.90 (16.59)	0.006
BMI at list (Kg/m^2)	Mean (SD)	27.55 (5.83)	27.67 (5.67)	27.46 (5.96)	0.64	27.85 (5.76)	28.20 (5.75)	27.52 (5.78)	0.24
Comorbidities	HTN	154 (22%)	061 (20%)	93 (24%)	0.16	035 (20%)	17 (20%)	18 (21%)	0.45
	DM	176 (26%)	059 (19%)	117 (31%)	<0.001	43 (25%)	9 (11%)	34 (39%)	<0.001
	Insulin Use	101 (15%)	36 (12%)	65 (17%)	0.05	20 (12%)	3 (4%)	17 (20%)	0.001
	Hyperlipidemia	89 (13%)	22 (7%)	67 (18%)	<0.001	19 (11%)	7 (8%)	12 (14%)	0.26
	CKD	36 (5%)	16 (5%)	20 (5%)	0.99	8 (5%)	3 (4%)	5 (6%)	0.72
	IHD	45 (7%)	7 (2%)	38 (10%)	<0.001	8 (5%)	2 (2%)	6 (7%)	0.28
Decompensations	Encephalopathy	506 (73%)	231 (75%)	275 (72%)	0.54	126 (74%)	63 (75%)	63 (72%)	0.70
	Variceal bleeding	282 (41%)	121 (39%)	162 (42%)	0.47	66 (39%)	28 (33%)	38 (44%)	0.16
	Ascites	596 (87%)	275 (90%)	321 (84%)	0.034	156 (91%)	80 (95%)	76 (87%)	0.07
	Paracentesis	344 (50%)	156 (51%)	188 (49%)	0.68	86 (50%)	43 (51%)	43 (49%)	0.82
	SBP	138 (20%)	66 (22%)	72 (19%)	0.39	40 (23%)	20 (24%)	20 (23%)	0.90
	HRS	156 (23%)	103 (34%)	53 (14%)	<0.001	38 (22%)	24 (29%)	14 (16%)	0.05
Na MELD (at listing)	Median (Range)	22 (6–54)	28 (13–54)	18 (6–33)	<0.001	22 (6–50)	28 (17–50)	18 (6–27)	<0.001
	<20	251 (36%)	013 (4%)	238 (62%)	<0.001	61 (36%)	4 (5%)	57 (66%)	<0.001
Na MELD (at end of listing)	Median (Range)	25 (6–57)	29 (6–57)	19 (7–46)	<0.001	24 (6–44)	27 (13–44)	19 (6–37)	<0.001
MDRD eGFR ml/min/1.73 m^2	Median (Range)	75 (15–120)	60 (15–120)	81 (15–120)	<0.001	73 (15–120)	69 (15–120)	83 (25–120)	<0.001
	<60	250 (36%)	149 (49%)	101 (27%)	<0.001	60 (35%)	37 (44%)	23 (26%)	0.02
Frailty score	Mean (SD)	4.23 (1.46)	4.15 (1.47)	4.29 (1.45)	0.28	4.14 (1.42)	4.18 (1.32)	4.10 (1.51)	0.76
	Moderate to severe	118 (23%)	41 (19%)	77 (25%)	0.07	27 (21%)	13 (21%)	14 (21%)	0.95
Blood group	A	257 (37%)	127 (41%)	130 (34%)	0.07	61 (36%)	26 (31%)	35 (40%)	0.16
	AB	48 (7%)	21 (7%)	27 (7%)		11 (6%)	7 (8%)	4 (5%)	

	B	90 (13%)	30 (10%)	60 (16%)		18 (11%)	6 (7%)	12 (14%)	
	O	293 (43%)	129 (42%)	164 (43%)		81 (47%)	45 (54%)	36 (41%)	
	AIH	39 (6%)	16 (5%)	23 (6%)		8 (5%)	5 (6%)	3 (3%)	
	CC	29 (4%)	18 (6%)	11 (3%)		5 (3%)	1 (1%)	4 (5%)	
	ALD	205 (30%)	99 (32%)	106 (28%)		52 (30%)	32 (38%)	20 (23%)	
	HBV	22 (3%)	15 (5%)	7 (2%)		10 (6%)	4 (5%)	6 (7%)	
Primary diagnosis	HCV	108 (16%)	45 (15%)	63 (16%)		21 (12%)	10 (12%)	11 (13%)	
	NASH	135 (20%)	58 (19%)	77 (20%)		41 (24%)	17 (20%)	24 (28%)	
	PBC	48 (7%)	14 (5%)	34 (9%)		8 (5%)	2 (2%)	6 (7%)	
	PSC	71 (10%)	27 (9%)	44 (12%)		16 (9%)	10 (12%)	6 (7%)	
	Others	32 (5%)	15 (5%)	17 (4%)		10 (6%)	3 (4%)	7 (8%)	
Etiology group	NASH	135 (20%)	58 (19%)	77 (20%)	0.68	41 (24%)	17 (20%)	24 (28%)	0.26
ICU stay in last 90 days	Y	117 (17%)	79 (26%)	38 (10%)	<0.001	143 (84%)	66 (79%)	77 (89%)	0.08
Cumulative LOS in last 90 days	Median (Range)	1 (0–90)	9.0 (0–90)	1.0 (0–90)	<0.001	1 (0–90)	3 (0–90)	1 (0–48)	<0.001
Number of hospitalizations in last 90 days	Median (Range)	1 (0–11)	1 (0–8)	1 (0–11)	<0.001	1 (0–16)	1 (0–14)	1 (0–16)	0.17
Time on waitlist (Days)	Median (Range)	101.0 (1.0–1865.0)	28 (1–1725)	228 (2–1865)	<0.001	136 (0–1903)	32 (0–1903)	286 (34–1656)	<0.001
Time to receive transplant (Days)	Median (Range)	74.0 (1.0–1725.0)	25 (1–1725)	152 (2–1511)	<0.001	75 (0–1566)	17.5 (0–706)	168 (38–1566)	<0.001
pLD	Yes	287 (42%)	104 (34%)	183 (48%)	<0.001	73 (43%)	35 (42%)	38 (44%)	0.79
	Waitlist mortality/ drop out	217 (31%)	85 (28%)	132 (35%)		49 (27%)	27 (32%)	22 (25%)	
Outcome type	Transplant/medical improvement	454 (66%)	219 (71%)	235 (62%)	0.005	119 (70%)	56 (67%)	63 (72%)	0.55
	Active listing/ care transferred	18 (3%)	3 (1%)	15 (4%)		3 (2%)	1 (1%)	2 (2%)	
	Active Listing	17 (2%)	2 (1%)	15 (4%)		3 (2%)	1 (1%)	2 (2%)	
	De-listed	117 (17%)	20 (7%)	97 (25%)		30 (18%)	4 (5%)	26 (30%)	
Outcome	Died	158 (23%)	73 (24%)	85 (22%)	<0.001	39 (23%)	25 (30%)	14 (16%)	<0.001
	Transplant	397 (58%)	212 (69%)	184 (48%)		99 (58%)	54 (64%)	45 (52%)	
Type of LT	DDLT	259 (65%)	179 (84%)	80 (43%)	<0.001	67 (68%)	41 (76%)	26 (58%)	0.05
	LDLT	138 (35%)	033 (16%)	105 (57%)		32 (32%)	13 (24%)	19 (42%)	

Abbreviations: AIH: Autoimmune hepatitis; ALD: Alcoholic liver disease; BMI: Body mass index; CC: Cryptogenic cirrhosis; CKD: Chronic kidney disease; DDLT: Deceased donor liver transplant; DM: Diabetes mellitus; eGFR: Estimated glomerular filtration rate; HBV: Hepatitis B virus; HCV: Hepatitis C virus; HRS: Hepatorenal syndrome; HTN: Hypertension; ICU: Intensive care unit; IHD: Ischemic heart disease; LDLT: Living donor liver transplant; LOS: Length of stay; LT: Liver transplant; NASH: Non-alcoholic steatohepatitis; PBC: Primary biliary cholangitis; pLD: Potential living donor; PSC: Primary sclerosing cholangitis; SBP: Spontaneous bacterial peritonitis; SD: Standard deviation.

Supplementary Table 3. Benefit of pLD in waitlisted cirrhosis patients stratified by risk factors.

Risk factors	All patients HR (95% CI)	Interaction *P* value	NASH HR (95% CI)	Interaction *P* value	Non-NASH HR (95% CI)	Interaction *P* value
Etiology NASH	1.59 (1.09–2.31) *P* = 0.026	0.35	–	–	–	–
Etiology Non-NASH	1.84 (1.51–2.25) *P* < 0.0001		–		–	
Age >60	1.88 (1.37–2.59) *p* = 0.0002	0.74	1.74 (1.01–3.0) *p* = 0.02	0.54	1.94 (1.31–2.87) *p* = 0.002	0.75
Age <60	1.77 (1.43–2.19) *p* < 0.0001		1.45 (0.87–2.41) *p* = 0.26		1.8 (1.43–2.28) *p* < 0.001	
Gender Female	2.02 (1.51–2.71) *P* < 0.0001	0.27	1.24 (0.70–2.20) *P* = 0.49	0.29	2.33 (1.65– 3.28) *P* < 0.0001	0.07
Gender Male	1.66 (1.33–2.08) *P* < 0.0001		1.91 (1.16–3.13) *P* = 0.02		1.59 (1.24–2.05) *P* = 0.001	

Height <160	1.68 (1.39–2.03)<br>*P* < 0.0001	0.03	2.48 (0.94–6.55)<br>*P* = 0.0725	0.34	3.37 (1.84–6.17)<br>*P* < 0.0001	0.04
Height >160	3.11 (1.85–5.22)<br>*P* < 0.0001		1.48 (0.98–2.22)<br>*P* = 0.0986		1.27 (1.39 2.12)<br>*P* < 0.0001	
eGFR<br>>60	2.18 (1.74–2.74)<br>*P* < 0.0001	0.04	2.07 (1.24–3.44)<br>*P* = 0.009)	0.23	2.20 (1.77–2.83)<br>*P* < 0.0001	0.11
eGFR<br><60	1.33 (1.00–1.78)<br>*p* = 0.08		1.20 (0.7–2.04)<br>*P* = 0.61		1.34 (0.95–1.90)<br>*P* = 0.12	
MELD<br><20	4.82 (3.34–6.96)<br>*P* < 0.0001	<0.0001	2.8 (1.44–5.42)<br>*P* = 0.006	0.21	5.62 (3.64–8.68)<br>*P* < 0.0001	<0.0001
MELD<br>>20	1.4 (1.14–1.72)<br>*P* = 0.0048		1.43 (0.91–2.23)<br>*P* = 0.16		1.39 (1.10–1.74)<br>*P* = 0.017	
BMI <30	1.86 (1.5–2.3)<br>*P* < 0.001	0.44	1.43 (0.81–2.53)<br>*P* = 0.30	0.92	1.92 (1.53–2.42)<br>*P* < 0.0001	0.46
BMI ≥30	1.64 (1.19–2.25)<br>*P* = 0.003		1.66 (1.01–2.71)<br>*P* = 0.04		1.57 (1.03–2.39)<br>*P* = 0.04	
Diabetes<br>Yes	2.14 (1.49–3.07)<br>*P* < 0.0001	0.36	2.32 (1.37–3.92)<br>*P* = 0.002	0.056	1.98 (1.22–3.22)<br>*P* = 0.008	0.74
Diabetes<br>No	1.70 (1.39–2.08)<br>*P* < 0.0001		1.05 (0.62–1.79)<br>*P* = 0.95		1.81 (1.45– 2.25)<br>*P* < 0.0001	
IHD<br>Yes	3.06 (1.32–7.06)<br>*P* = 0.003	0.3	2.35 (0.81–6.85)<br>*P* = 0.08	0.5	3.84 (1.00–12.16)<br>*P* = 0.04	0.36
IHD<br>No	1.76 (1.47–2.11)<br>*P* < 0.0001		1.51 (1.01–2.26)<br>*P* = 0.07		1.81 (1.47–2.21)<br>*P* < 0.0001	
Frailty<br>More	2.76 (1.64–4.64)<br>*P* < 0.0001	0.03	1.97 (0.82–4.7)<br>*P* = 0.20	0.44	3.11 (1.64–5.92)<br>*P* = 0.0003	0.03
Frailty<br>Less	1.67 (1.33–2.09)<br>*P* < 0.0001		1.49 (0.95–2.32)<br>*P* = 0.11		1.69 (1.30–2.20)<br>*P* = 0.0001	
Prediction<br>Score High	4.08 (2.96–5.6)<br>*P* < 0.001	<0.0001<br>Derivation set	3.15 (1.65–6.03)<br>*P* = 0.0007<br>(*n* = 77)	0.19<br>Derivation set	4.28 (2.97–6.18)<br>*P* < 0.0001<br>(*n* = 305)	<0.0001<br>Derivation set
Prediction<br>Score Low	1.02 (0.79–1.32)<br>*P* = 0.82		1.39 (0.77–2.50)<br>*P* = 0.32<br>(*n* = 58)		0.95 (0.71–1.26)<br>*P* = 0.48<br>(*n* = 249)	
Prediction<br>Score High	3.83 (2.10–6.99)<br>*P* < 0.001	0.05<br>Validation set	1.33 (0.56–3.16)<br>*P* = 0.74<br>(*n* = 24)	0.21<br>Validation set	6.15 (2.68–14.10)<br>*P* < 0.0001<br>(*n* = 63)	0.06<br>Validation set
Prediction<br>Score Low	1.26 (0.75–2.12)<br>*P* = 0.40		0.52 (0.16–1.68)<br>*P* = 0.22<br>(*n* = 17)		1.65 (0.93–2.92)<br>*P* = 0.10<br>(*n* = 67)	

Abbreviations: BMI: Body mass index; GFR: Glomerular filtration rate; IHD: Ischemic heart disease; MELD: Model for end stage liver disease; NASH: Non-alcoholic steatohepatitis; pLD: Potential living donor.
